# Congenital Varicella Syndrome and Crossed Nonfused Renal Ectopia in a Neonate: A Case Report

**DOI:** 10.7759/cureus.94368

**Published:** 2025-10-11

**Authors:** Neha Jain, Vivek Goyal, Sreevidya Sreekantha

**Affiliations:** 1 Pediatrics and Neonatology, Janki Children Hospital, Hisar, IND; 2 Pediatrics and Neonatology, Motherhood Women and Children's Hospital, Bengaluru, IND

**Keywords:** cicatricial skin lesions, congenital varicella syndrome, crossed nonfused renal ectopia, hypoxic-ischemic encephalopathy, maternal varicella

## Abstract

Congenital varicella syndrome (CVS) is a rare consequence of maternal varicella infection during early pregnancy and is characterized by cicatricial skin lesions, neurological impairment, limb hypoplasia, and ocular abnormalities. Renal anomalies are not classical features but may occur. We report a term male neonate, born to a primigravida mother with a history of varicella infection at 12 weeks of gestation. The neonate presented with perinatal asphyxia, seizures, cicatricial skin lesions, and a rare renal anomaly - crossed nonfused renal ectopia (CNRE). The neonate required resuscitation at birth, developed hypoxic-ischemic encephalopathy, and was managed with therapeutic hypothermia and antiepileptics. Radiological imaging revealed CNRE. Both maternal and neonatal varicella IgG serology were positive. The baby improved with supportive management and was discharged in stable condition. To the best of our knowledge, CVS and CNRE are not correlated and represent coincidental, unrelated findings in our case.

## Introduction

Varicella zoster virus (VZV), a member of the herpesvirus family, causes varicella (chickenpox) as the primary infection and herpes zoster (shingles) upon reactivation. Varicella is a common childhood illness characterized by fever and a generalized pruritic vesicular rash, while herpes zoster manifests as a localized, painful vesicular eruption involving one or adjacent dermatomes [1].

Maternal varicella infection during pregnancy, while uncommon, represents a potentially grave condition associated with significant fetomaternal morbidity and mortality. The clinical manifestations of gestational varicella infection are temporally dependent, with distinct outcomes correlating to the timing of viral acquisition. Varicella infection in the first 20 weeks of pregnancy can lead to congenital varicella syndrome (CVS), while varicella infection in the third trimester can result in maternal pneumonia. The risk of disseminated varicella infection in the neonate, known as neonatal varicella, is highest if the mother acquires the infection between five days antepartum to two days postpartum [2].

Clinical features of CVS include characteristic cicatricial skin scarring with a dermatomal distribution, ocular abnormalities (cataracts, microphthalmia, and chorioretinitis), limb hypoplasia (usually ipsilateral to scarring), and neurological abnormalities (cortical atrophy, seizures, microcephaly, and intellectual disability). Autonomic nervous system dysfunction can also occur, resulting in neurogenic bladder, esophageal dilation, and gastroesophageal reflux [3,4].

Renal anomalies such as dysplasia, hypoplasia, and hydronephrosis are uncommon manifestations of CVS. Crossed nonfused renal ectopia (CNRE) is an exceedingly rare condition, and to the best of our knowledge, no previous reports have documented its detection during the neonatal period. In our case, CVS and CNRE appear to be unrelated conditions, representing coincidental findings.

## Case presentation

A term small-for-gestational-age (SGA) male neonate, with a birth weight of 2.5 kg, was delivered at 41 weeks of gestation via normal vaginal delivery to a 22-year-old primigravida mother. The antenatal period was complicated by pregnancy-induced hypertension and prolonged rupture of membranes lasting more than 18 hours.

The baby exhibited a delayed onset of spontaneous respiration, requiring brief bag-and-mask ventilation. Progressive respiratory distress necessitated referral to our tertiary neonatal intensive care unit at two hours of life (HOL) on supplemental oxygen support via nasal prongs.

**Figure 1:**
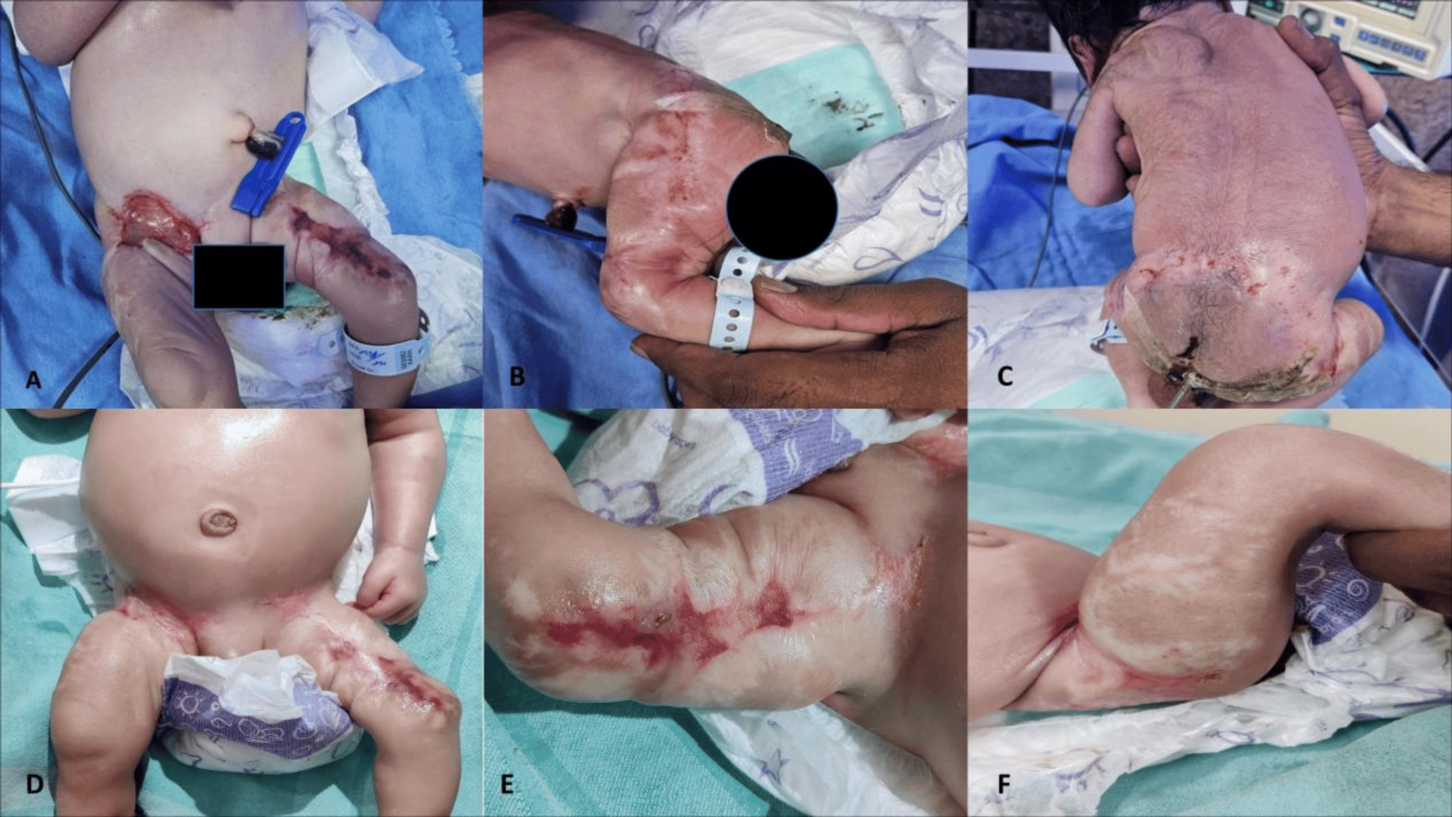
Serial clinical photographs of the neonate showing cicatricial rash over both thighs, groin, and lower back. Panels A-C were taken on DOL 1, and panels D-F were taken on DOL 4. (A) Cicatricial lesions over both thighs and groin on DOL 1, corresponding to the L1-L4 lumbar dermatomes. (B) Cicatricial scarring over the lateral aspect of the left thigh, corresponding to the L5 dermatome. (C) Extensive cicatricial lesions involving the lower back, corresponding to the L5 dermatome. (D) Healed cicatricial lesions over the thighs and groin on DOL 4. (E) Linear cicatricial scarring over the anterior aspect of the left thigh. (F) Hypopigmented cicatricial lesion over the lateral aspect of the right thigh. DOL: day of life

On admission, the baby was hypoxic (SpO_2_ 78%) with mild tachypnea (respiratory rate 64/min) and tachycardia (heart rate 160/min). Physical examination revealed mild subcostal and intercostal retractions, hypotonia, lethargy, and moderate encephalopathy as per modified Sarnat and Sarnat staging [5]. A cicatricial rash was noted over both thighs, groin, and lower back, involving the L1-L5 lumbar dermatomes (Figure 1). 

Additionally, a single umbilical artery (SUA) was observed. The baby had a length of 51 cm and an occipitofrontal circumference (OFC) of 34 cm, with no limb abnormalities noted.

The neonate developed multiple episodes of seizures by four HOL, requiring antiepileptic medications. Arterial blood gas analysis revealed severe metabolic acidosis (pH: 7.08; base deficit: -21). The baby was initiated on continuous positive airway pressure (CPAP) support and therapeutic hypothermia using a servo-controlled cooling device (CritiCool®, Belmont Medical Technologies, USA). Intravenous fluids, broad-spectrum antibiotics, and antiepileptics (phenobarbitone and levetiracetam) were administered. Initial laboratory parameters (Table 1) showed a negative septic screen, with normal renal function, electrolytes, and coagulation profile.

**Table 1:** Laboratory investigations. DOL: day of life; CRP: C-reactive protein; TLC: total leukocyte count; CSF: cerebrospinal fluid; SGOT: serum glutamic-oxaloacetic transaminase; SGPT: serum glutamic-pyruvic transaminase; NA: not applicable

Parameters	DOL 1	DOL 3	DOL 5	DOL 7	DOL 10	Reference values
Hemoglobin	17.8	15.8	16.1	17.0	15.8	12-16 g/dL
TLC	31.8	7.4	4.8	4.5	6.9	4-12 x 10^9 ^cells/L
Platelet count	196	143	107	116	161	150-450 x 10^9 ^cells/L
CRP	1.3	1.14	48.5	16.2	5.1	<6 mg/dL
Urea	NA	53	35.6	NA	NA	16-43 mg/dL
Creatinine	NA	1.33	0.70	NA	NA	0.3-1.3 mg/dL
Sodium	140	140	145	NA	NA	135-145 mmol/L
Potassium	4.65	3.2	5.4	NA	NA	3.5-5.5 mmol/L
Ionized calcium	4.9	3.8	4.7	NA	NA	4.5-4.9 mg/dL
SGOT	NA	129.8	NA	NA	NA	<35 U/L
SGPT	NA	73.9	NA	NA	NA	<45 U/L
Blood culture	No growth	NA	No growth	NA	NA	-
CSF TLC / glucose / proteins	NA	5 42.2 152.4	NA	NA	NA	0-30 cells/cu.mm / 50-80 mg/dL / 15-45 mg/dL
Varicella IgG	NA	NA	3347.0	NA	NA	<150 mIU/mL
Varicella IgM	NA	NA	<0.10	NA	NA	<0.10 index

Enteral nutrition was cautiously initiated on day of life (DOL) 2 with gradual advancement as tolerated. Seizures subsided by DOL 3 and did not recur. Cerebrospinal fluid analysis ruled out meningitis. Serial monitoring of renal function and coagulation profiles remained normal. 

Rewarming was initiated after 72 hours of cooling. On DOL 5, laboratory evaluation revealed a positive septic screen with thrombocytopenia but a sterile blood culture (culture-negative sepsis), which resolved with antibiotics.

**Figure 2:**
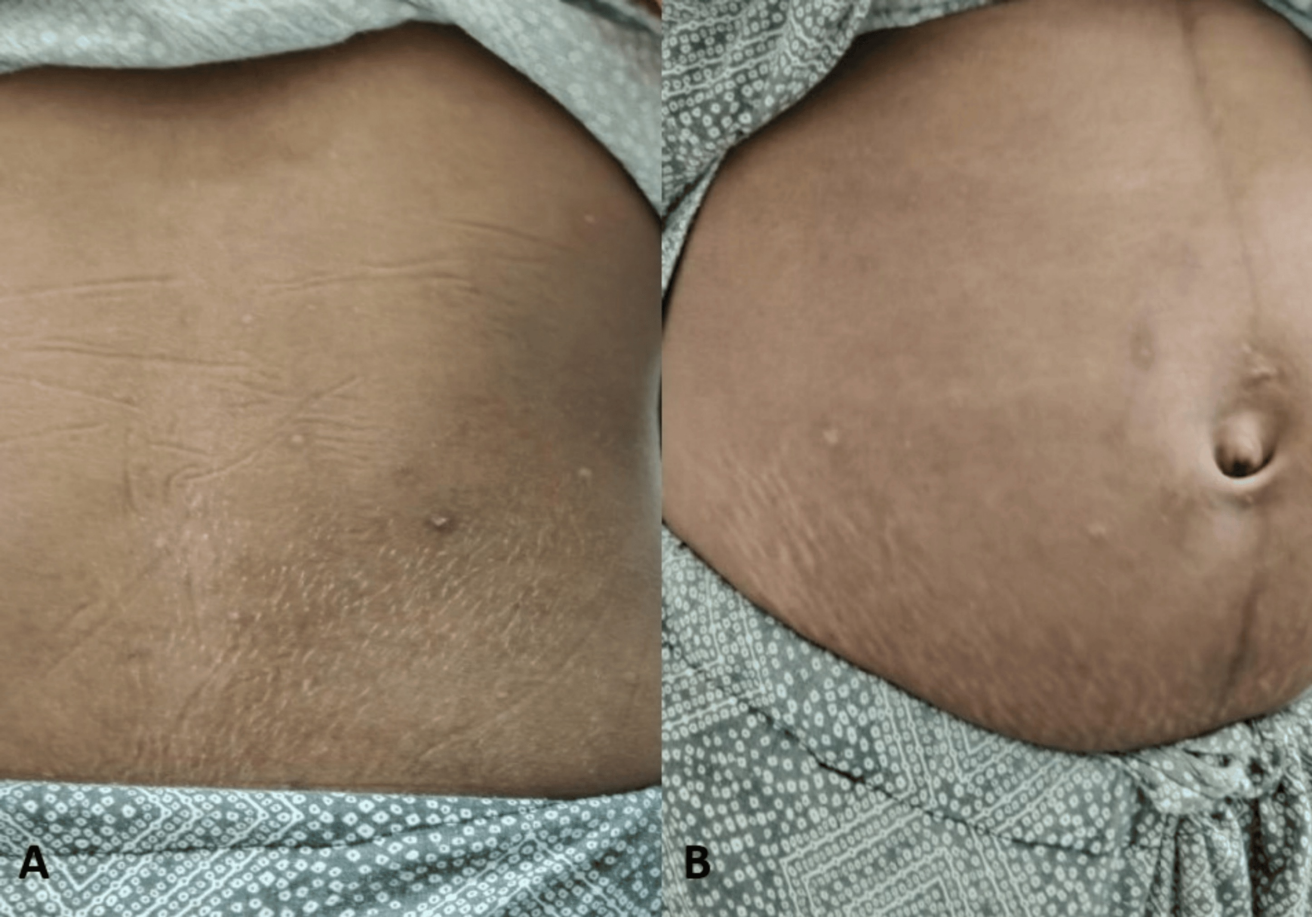
Photographs showing post-varicella cutaneous scarring in the mother. (A) Multiple hypopigmented scars over the back. (B) Hypopigmented scars over the abdomen.

Given the characteristic cicatricial rash, a detailed maternal history was revisited. The mother reported a febrile illness with rash at 12 weeks of gestation, and post-varicella scars were evident on examination (Figure 2).

**Figure 3:**
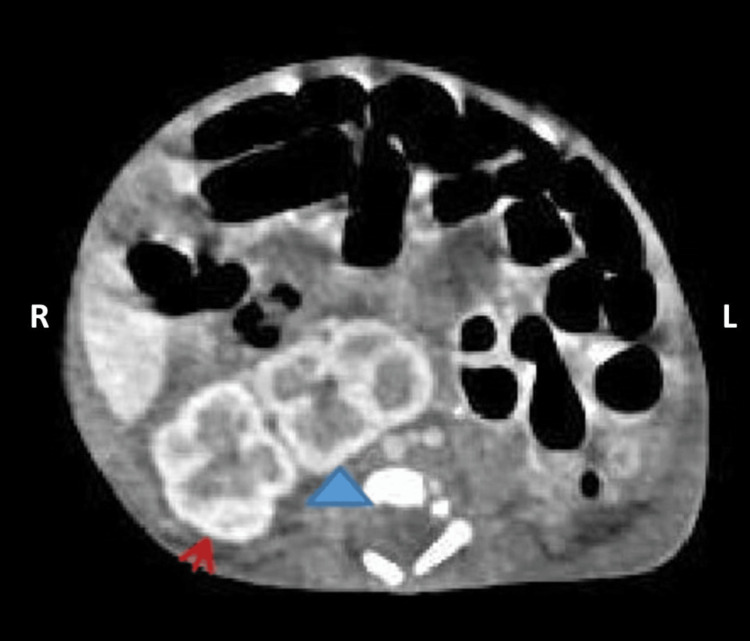
Axial contrast enhanced CT scan showing left crossed nonfused renal ectopia. The left kidney (blue arrowhead) is located in the right lumbar region, medial to the right kidney (red arrow), and is without fusion.

Varicella IgG serology was positive in both the mother and baby (3,745 and 3,347 mIU/mL, respectively), while IgM was negative. In view of SUA, abdominal ultrasonography was performed and revealed a left crossed ectopic kidney, which was subsequently confirmed by contrast-enhanced CT imaging as left CNRE (Figure 3). 

Cranial ultrasound and ophthalmological assessments were normal. Dermatological assessment excluded other possible etiologies for the skin lesions. 

Respiratory distress resolved by DOL 4, and the baby was weaned off CPAP and maintained adequate oxygen saturation on room air. The baby tolerated feeds well, was treated for clinical sepsis, and was discharged in stable condition on DOL 12.

## Discussion

Varicella is usually a mild and self-limiting illness in immunocompetent children, but it can pose significant health risks to vulnerable populations, including older adults, immunocompromised individuals, and pregnant women. Maternal VZV infection during pregnancy increases the risk of severe complications for both the mother and fetus. Maternal complications can include encephalitis and pneumonia, while fetal risks depend on the gestational age at the time of infection. Infection during the first half of pregnancy can result in CVS, whereas infection around the time of delivery may cause neonatal varicella, which can be severe and life-threatening [6].

CVS is estimated to occur in approximately 0.4% of infants when maternal varicella occurs between eight and 13 weeks of gestation and up to 2% of infants when infection occurs between 13 and 20 weeks [7]. There are a few case reports of infants with CVS following maternal infection between 21 and 28 weeks of gestation [8,9].

CVS is characterized by multisystem involvement, including dermatologic, neurologic, musculoskeletal, and ocular abnormalities. Skin lesions, seen in approximately 72-73% of cases, typically present as scarring in a dermatomal distribution, believed to result from viral damage to developing sensory nerves. Neurological abnormalities, such as microcephaly, cortical atrophy, hydrocephalus, and intellectual disability, occur in 48-62% of cases. Ocular abnormalities, such as microphthalmia, chorioretinitis, and cataracts, occur in 44-52% of cases, and limb hypoplasia occurs in 46-72%. Other systemic anomalies affecting the gastrointestinal, cardiovascular, and genitourinary systems occur in 12% of affected neonates [10].

In our case, the baby had cicatricial skin scarring in a dermatomal pattern (L1-L5) and neurological impairment in the form of encephalopathy and seizures. However, the neurological derangements were attributed to hypoxic-ischemic encephalopathy, as evidenced by the need for resuscitation at birth, acidosis in the first blood gas (pH: 7.08, base deficit: -21), onset of seizures on DOL 1, and moderate encephalopathy (stage 2 as per modified Sarnat and Sarnat staging). The seizures subsided by DOL 3 and neurological recovery was observed, further supporting a primary hypoxic etiology rather than direct VZV-related brain injury.

Diagnosis of CVS was made in view of the presence of characteristic cicatricial rash in the L1-L5 dermatomal pattern, coupled with maternal history of varicella infection at 12 weeks of gestation and high VZV IgG titres in both the mother and baby.

A unique finding in our case was the presence of CNRE, a rare congenital anomaly in which both kidneys occupy the same side of the body while one ureter traverses across the midline to graft into the contralateral bladder. McDonald and McClellan have categorized this condition into four subtypes: (i) crossed renal ectopia with fusion; (ii) crossed renal ectopia without fusion; (iii) simple cross-fused renal ectopia; and (iv) bilaterally crossed renal dystopia [11,12].

Crossed fused renal ectopia is reported in approximately one in 7,500 autopsies, whereas CNRE is about ten times rarer, occurring in one out of 75,000 autopsies [13]. It is typically asymptomatic and detected incidentally during imaging.

The precise embryological cause of crossed ectopia has not been identified. Several theories have been postulated, including the influence of genetic or teratogenic factors, as well as malalignment and abnormal rotation of the caudal end of the embryo, which may lead to aberrant development of the metanephric blastema and ureteric bud during the fourth to eighth week of gestation [14].

Importantly, crossed renal ectopia is not a recognized or classical feature of CVS, and a review of the existing literature does not reveal any established association between the two conditions. While genitourinary anomalies, such as renal hypoplasia and hydronephrosis, have occasionally been reported in CVS, the occurrence of crossed renal ectopia in our patient does not represent a direct manifestation of VZV teratogenicity [15].

## Conclusions

This case highlights that clinicians should maintain a high index of suspicion for CVS in neonates presenting with a characteristic cicatricial rash. A detailed maternal history of varicella infection during pregnancy should be obtained, and varicella serology should be performed in both the mother and infant. The presence of CNRE was an incidental finding. Additionally, when SUA is present, further evaluation should be undertaken to rule out congenital anomalies of the kidneys and urinary tract.
